# A Cost Analysis of Implementing a Blockchain Architecture in a Smart Grid Scenario Using Sidechains

**DOI:** 10.3390/s20030843

**Published:** 2020-02-05

**Authors:** Iago Sestrem Ochôa, Luis Augusto Silva, Gabriel de Mello, Nuno M. Garcia, Juan Francisco de Paz Santana, Valderi Reis Quietinho Leithardt

**Affiliations:** 1 Laboratory of Embedded and Distributed Systems-LEDS, University of Vale do Itajaí, Itajaí, SC 88302-901, Brazil; luis.silva@edu.univali.br (L.A.S.); gabrieldemello@edu.univali.br (G.d.M.); valderi.leithardt@ubi.pt (V.R.Q.L.); 2 Departamento de Informática e Redes de Computadores, Instituto Federal Catarinense (IFC), Brusque 88354-300, Brazil; 3 Expert Systems and Applications Lab, Faculty of Science, University of Salamanca, Plaza de los Caídos s/n, 37008 Salamanca, Spain; fcofds@usal.es; 4 Departamento de Informática, Universidade da Beira Interior, 6201-001 Covilhã, Portugal; ngarcia@di.ubi.pt; 5 Instituto de Telecomunicações, Universidade da Beira Interior, 6201-001 Covilhã, Portugal; 6 COPELABS, Universidade Lusófona de Humanidades e Tecnologias, 1749-024 Lisboa, Portugal

**Keywords:** blockchain, sidechain, smart grid

## Abstract

Smart grid systems have become popular and necessary for the development of a sustainable power grid. These systems use different technologies to provide optimized services to the users of the network. Regarding computing, these systems optimize electrical services by processing a large amount of the data generated. However, privacy and security are essential in this kind of system. With a large amount of data generated, it is necessary to protect the privacy of users, because this data may reveal the users’ personal information. Today, blockchain technology has proven to be an efficient architecture for solving privacy and security problems in different scenarios. Over the years, different blockchain platforms have emerged, attempting to solve specific problems in different areas. However, the use of different platforms fragmented the market, which was no different in the smart grid scenario. This work proposes a blockchain architecture that uses sidechains to make the system scalable and adaptable. We used three blockchains to ensure privacy, security, and trust in the system. To universalize the proposed solution, we used the Open Smart Grid Protocol and smart contracts. The results show that architecture security and privacy are guaranteed, making it feasible for implementation in real systems; although scalability issues regarding the storage of the data generated still exist.

## 1. Introduction

A Smart Grid (SG) is a large-scale electrical network infrastructure mainly characterized by security, agility, and resilience, which is capable of handling new threats and unforeseen conditions. In 2005, the authors introduced this concept in [1], known as smart electrical networks. The agents that act on these networks can communicate and cooperate in a self-configuring mode, in which they can consider whether a new element can join the network, or if a random event can requires correction. Although SG networks ensure efficiency in electrical systems, problems still exist for their implementation to be efficient in a holistic way.

According to [2], one of the problems to be solved for the implementation of SG networks is the privacy issue. The work developed in [3] states that, in general, data privacy affects the security of those connected, as a result of stored information related to the user’s life. The use of access control techniques, which guarantee reliable authentication, authorization, and confidentiality of the services, does not ensure a holistic solution to the privacy problem. This difficulty arises because the data needs to be disseminated in different parts of the network. In addition to privacy issues, another challenge facing the popularization of SG networks is security. Security problems on SG networks can cause disastrous effects on the network. According to [4], an SG network is vulnerable to cyber-attacks such as traffic analysis, social engineering, cracking, spoofing, denial of service, and others. If a security flaw exists in the equipment connected to the network, which could compromise the system, an update would be necessary to correct all devices, incurring a high monetary cost. To avoid security problems, the use of communication protocols that guarantee security in SG networks is essential.

For the data monitoring and communication in SG networks, different protocols exist for these areas. According to [5], protocols aim to ensure efficient solutions to the reliability and security of the network. However, the use of different protocols fragments the development of new applications, generating different network architectures directed to the SG segment. The use of a protocol that satisfies the requirements of an SG network is necessary for the development of new applications.

The Open Smart Grid Protocol (OSGP) is a protocol widely used in SG applications. OSGP Alliance developed the OSGP, which is published as a standard by the European Telecommunications Standards Institute [6]. The protocol implements all layers of the OSI model and provides security through cryptographic methods for Smart Meters (SM). However, studies expose security flaws on the OSGP encryption method. The work presented by Kursawe and Peters [7] shows a structural weakness in the cryptographic process of the OSGP. The main flaw observed was the use of RC4 encryption: with each new key generated for each message transmitted, only the first eight bytes of this new key is different from the others. Another problem observed was that only one key is used for authentication. This same authentication key is used to derive the encryption key, so if the authentication key is exposed, all encryption keys are compromised.

Security flaws are not exclusive of the OSGP protocol. As shown in [5], other SG protocols also have security flaws that can compromise the entire network. Conventional techniques of privacy and security are not sufficient to guarantee these requirements. For this, it is necessary to use an architecture that holistically guarantees security and privacy.

In 2008, Satoshi Nakamoto presented the Bitcoin system to the world. Bitcoin is a virtual currency, also known as cryptocurrency [8]. This technology works based on P2P communication among network users, eliminating the need for a third party to validate transactions between the peers of the network. To ensure integrity, security, privacy, and reliability of the data transmitted over the network, Bitcoin uses the technology known as the blockchain. The blockchain acts as a distributed reason book. The information is stored on blocks and validated through a consensus algorithm. The process of validating blocks is called mining. To encourage users to participate in the mining process, users that participate in the mining process receive a reward in cryptocurrency. As a result of its characteristics, the blockchain caught the attention of the application’s developers.

Blockchain proved to be an innovative technology due to its characteristics, which can solve security and privacy issues [9]. It is possible to find blockchain usage in medical environments [10], in IoT scenarios [11], and in industrial environments [12]. The trend of blockchain use was not different for the SG scenario. Commercial solutions that use blockchain technology in SG scenarios already exist. Nowadays, the primary use of this technology consists of electrical energy trade between different consumers. However, different works are attempting to develop blockchain architectures for the SG scenario that guarantee user security and privacy.

With the development of blockchain architectures focused on the SG scenario, various authors propose entirely new solutions that lack the use of existing SG protocols. These solutions are difficult to implement as a result of the complexity of adapting them to devices that already exist in SG networks. The use of existing protocols on new blockchain architectures can favor the implementation of this technology in SG networks.

To solve the problems previously presented, in this article, we propose a blockchain architecture focused on the SG scenario that uses sidechains. Our architecture uses the OSGP protocol integrated into three different blockchains, and proposes to guarantee privacy and security in SG networks holistically. Our architecture allows users to define their privacy preferences in a tamper-proof way, using a privacy blockchain. The electric company’s access to the information of each user is stored in a storage blockchain to ensure the reliability of the system. In this way, users and companies benefit from the use of this type of architecture.

The principal contribution of this article is the definition of a blockchain architecture that uses a protocol widely used in the SG scenario, supporting the implementation of this architecture on existing systems. Regarding the functionalities of our architecture, it provides security, reliability, and privacy for users through the use of different blockchains. Our architecture also provides scalability for SG applications, implementing the architecture in a sidechain concept, specifically designed to enable large-scale application development using the OSGP protocol.

The paper is structured as follows. Section 2 presents the background with the fundamental concepts necessary to understand this work. Section 3 shows the related works. Section 4 demonstrates the methodology used in the development of the proposed architecture and the details of our architecture. Section 5 illustrates the results obtained through the tests developed. Finally, Section 6 shows the conclusions obtained with the development of this work.

## 2. Background

In this section, the fundamental concepts necessary to understand our architecture are presented. In Section 2.1, we present the SG concept focusing on security and privacy issues. We also show the blockchain concept as a solution to security and privacy problems in Section 2.2. In Section 2.3, we outline the advantages of using the Ethereum blockchain in application development, the concept of sidechain, and how it can revolutionize blockchain technology.

### 2.1. Smart Grid

According to [13], an SG system is the integration of information technology with the generation, transmission, and distribution systems of electrical energy. It is possible to describe four characteristics of an SG system: (i) Increased efficiency and profitability of the system; (ii) the supply of tools for the consumer to manage energy use; (iii) the optimization of the resilience and quality of energy of the system; (iv) the development of new technologies such as renewable energy generation (solar, wind, and others), storage of energy (batteries), and electric vehicles.

One of the critical features in an SG network is that consumers also become producers (or prosumers); this happens because they can produce renewable energy in their houses through alternative sources. Analyzing this from an extended perspective, they acquire the responsibility of generating electricity with the same quality of traditional generation sources. According to the essential characteristics of the SG networks mentioned previously, with decentralized prosumers, three of these characteristics are guaranteed [14].

To ensure efficient management of energy usage, SG networks need to allow the prosumers to perform real-time monitoring of electricity consumption and generation. In this way, they can choose to store or sell the energy excess produced for other SG network users. SG networks require the development of communication infrastructures that support the growth and density of the system, guaranteeing the quality of the service, which is necessary for the operation in large scale applications.

The Smart Meter (SM) is the critical part of an SG network. An SM is responsible for collecting, processing, and managing the information obtained about the electrical usage of a residence. They are also responsible for collecting data from the electricity grid. The functionalities of an SM are various: these functionalities are intended to provide the consumer with a wide range of information, such as the amount of energy consumed in real time, the amount of energy used in the last hour, week, and month (and how much it cost), the use of electricity classified into high, medium, or low levels, amongst others. With the amount of information obtained from the user through an SM, privacy issues appear [15,16].

According to [17], even when transporting the data obtained by the SM in a secure communication channel to the electrical company, the electrical company still receives a large amount of data. With this data, it is possible to know when the consumer is at home, as shown in [18]. Some devices have specific consumption patterns, so it is possible to determine, for example, when television or a washing machine is turned on.

Regarding security, failures can seriously compromise network performance. One example of what can happen as a result of a security breach in SG systems is a fraud related to the SMs. If a hacker has access and controls the information contained in an SM, it is possible to manipulate metering data and send forged data to the electrical company. Considering a catastrophic scenario, a hacker could have full access and control over someone’s SM [19].

On the basis of the information presented, security and privacy are two critical points in SG systems. Thus, it is necessary to ensure these requirements holistically for SG systems to operate efficiently. For this, the use of different technologies can help in the development of architectures that guarantee security and privacy in SG environments.

### 2.2. Blockchain

Proposed by Nakamoto in 2008 [8], Bitcoin proposes to promote the exchange of its cryptocurrency in a decentralized way using the blockchain technology to store the data and guarantee the authenticity of the transactions made. Over the years, blockchain technology has caught the attention of researchers because it can enable the development of various applications besides the exchange of cryptocurrencies between users.

The blockchain is a technology with the primary function of guaranteeing information security by storing transactions in a decentralized way between the nodes of the network. The blockchain can be compared to a public ledger, creating consensus and direct communication between two parties, without the intermediary of third parties.

**Figure 1:**
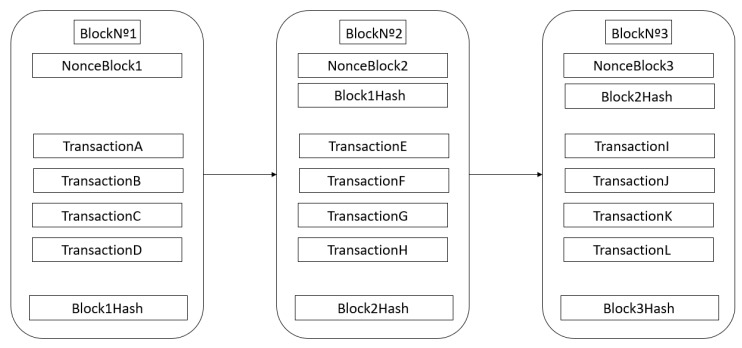
Blockchain structure.

Transactions consist of information stored inside blocks that are later validated through a consensus algorithm and added to the blockchain. The transactions performed are stored within the blocks: the hash of the current block, the hash of the previous block, the number of the last block, and a nonce value. Figure 1 illustrates the blockchain structure described.

The information stored in the blockchain refers to transactions performed by network users. The hash consists of encrypting all the information stored in the blocks. The hash of the previous block links one block to another, thus ensuring the integrity of the entire chain. The block number corresponds to the identification of that block in the blockchain. Finally, the objective of the nonce is as a unique number for the hash function, that is, to be able to generate the hash of a block; the miners, responsible for the creation of the blocks, must hit the nonce value to obtain the correct hash value.

With the popularization of blockchain technology, developers began to create their own blockchains, each with its purpose and particular characteristics. One of the most innovative blockchains developed is the Ethereum platform, also known as the Ethereum network.

Ethereum is a platform able to execute Smart Contracts (SC) and store them in a blockchain. Contracts executed on the Ethereum platform are immutable and work precisely as programmed, without any possibility of changing the code after it is created and stored in the blockchain. Ethereum was established in 2014 by Vitalik Buterin through a crowdfunding project [20].

**Figure 2:**
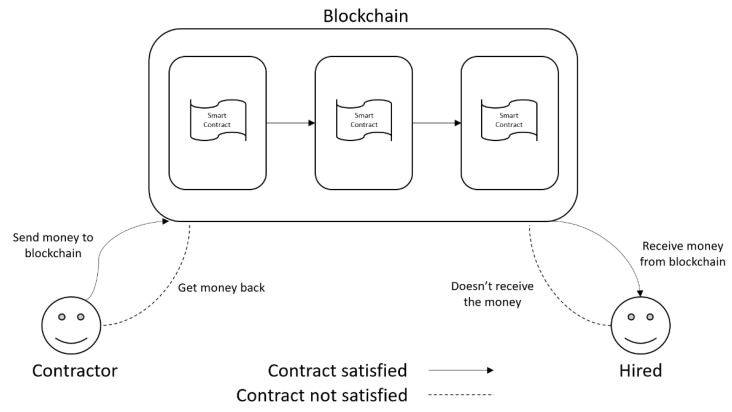
Smart contract operation.

Figure 2 illustrates the execution of smart contracts in the Ethereum platform. Considering the scenario whereby a person intends to finance a project, the rule of the contract is that the payment is released to the project developer only when the project is completed and working. If this condition is not satisfied, the contractor receives the money back. As seen in Figure 2, SC can ensure that both parties are satisfied in each case of contract operation.

The consensus algorithm used to mine new blocks in the Ethereum blockchain is known as EtHash Proof-of-Work (PoW). This algorithm was proposed to solve the ASIC mining problem of the Bitcoin consensus algorithm, see [21,22]. However, it is intended to migrate the consensus algorithm of the Ethereum network to the Proof-of-Stake (PoS). In the PoS algorithm, a random number generator guided by the amount of cryptocurrency that users own determines the miner of the next block [23].

To encourage network participants to mine new blocks, the Ethereum network has a cryptocurrency called Ether, denoted by the ETH pseudonym. The Ethereum white paper documents the denominations of each part of the Ethereum cryptocurrency [24].

Different references show the use of the Ethereum platform for the development of applications in different areas. In [25,26], the authors developed implementations to ensure privacy in IoT environments. In [27], the authors developed a blockchain architecture to prevent fake news in social media. In [28], the authors used the Ethereum platform to optimize agricultural services. In [29], the authors described a blockchain architecture for charging electric vehicles. In [30], the use of the Ethereum network for healthcare systems is presented.

With the development of new applications due to platform popularity, in January 2018, the Ethereum network registered a peak of approximately 1.25 million transactions made in its blockchain [31]. It is necessary to mention that a value called the gas limit determines the limit of transactions per block in the Ethereum platform. The gas limit value is defined by the users who mine new blocks in the Ethereum network. Considering that the Ethereum network has been dramatically increasing in size due to its popularity, scalability issues also appear with the development of new applications. The processing of transactions that operate on the Ethereum platform can be affected because of the large number of transactions processed at the same time. To solve the scalability problem, it is necessary to use blockchains that work in parallel with each other. This concept is known as a sidechain.

### 2.3. Sidechain

Sidechain is a type of blockchain that validates data from other blockchains. This technology has been developed to avoid fragmentation of existing markets. Since the creation of Bitcoin, different blockchains have been created, thus fragmenting the market. Sidechains allow integration between blockchains, without modifying the basic scripts of existing blockchains [32].

**Figure 3:**
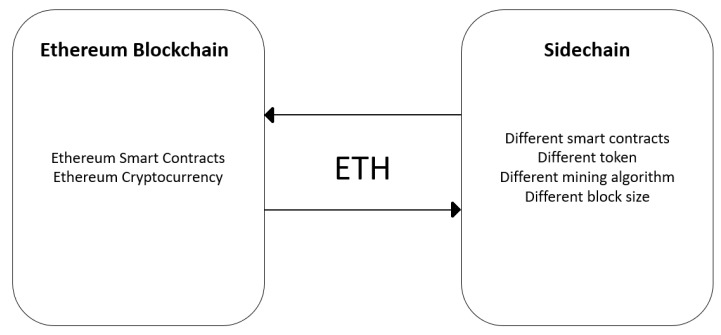
Sidechain concept.

According to [33], sidechains must satisfy the following requirements: Cryptocurrency moved between sidechains must be able to be retrieved by whomever the owner is. Transfers must be binary (e.g., happen or not happen; there should not be failures that create cryptocurrency fragmentation). If there is a bug in a sidechain, this bug cannot interfere in another sidechain. The sidechains must be independent. Finally, users should not need to find sidechains that they are not actively using. Figure 3 illustrates the situation described in this paragraph.

The decentralized validation process, known as pegging, allows cryptocurrencies to be imported from a blockchain and returned to other blockchains. Pegging is a symmetric validation process; to transfer cryptocurrency from a blockchain to a sidechain, it must be sent to an output address in the main blockchain and can be unlocked through a sidechain work test. Pegging can also be asymmetrical. In this process, the sidechain users are miners of the main blockchain, the transfers between the main blockchain and the sidechain do not require a PoW method [32].

Another type of validation for transactions between blockchains and sidechains uses a method called federation. The federation is an intermediate layer between blockchains and sidechains whose function is to manage the users’ cryptocurrency transactions. The owner of the sidechain chooses the members of the federation.

Regarding security, each sidechain is responsible for securing its network. In cases where a failure compromises the security of the sidechain, this failure must not affect the main blockchain. However, if a security flaw compromises the main blockchain, the sidechain still works, but the pegging method loses its value.

Sidechain is a new technology, but it offers numerous advantages in the development of decentralized applications. The interaction between different cryptocurrencies occurs through the sidechain architecture. Furthermore, when a sidechain exists for a determined purpose of operation, it is not necessary to create another sidechain with the same functionality. Finally, sidechains help to improve the scalability of the system since transactions made in sidechains can be processed independently of the main blockchain.

## 3. Related Work

This section presents the related works. State-of-the-art solutions are presented to support the architecture proposed in this article.

Guan et al. [34] propose an architecture that divides users into groups, where each group has its private blockchain, and each user is associated with a pseudonym to disguise their identity. The authors use the bloom filter to validate aliases and check for fake users. This way, an attacker would only know the sum of the group’s electricity consumption, without knowing each user’s data. Using aliases and encryption ensures the privacy of network users. However, the authors do not specify the platform and communication protocol used.

Gür et al. [35] describe a blockchain-based system for metering and billing with privacy protection. According to the authors, the decentralized blockchain architecture and cryptographic algorithms ensure data privacy and security of the network. The authors used the Hyperledger platform because it has an architecture that allows blockchain construction in a modular way. To simulate the smart meters, they used the Raspberry Pi 3 to generate random measurements. In the proposed architecture, the authors manage privacy by keeping data on devices, being shared only when necessary, and using encryption in communication, although the authors do not address the communication protocol or the cryptographic algorithm used.

Gai et al. [36] proposed an architecture for solar panels. The authors used blockchain to ensure reliability in energy trade among users. To avoid storage vulnerabilities, the authors implemented a distributed ledger. To ensure privacy, they used a method of account creation based on the user’s energy use. The proposed implementation uses the Hyperledger platform. A private blockchain was developed, and comparisons were made with a public blockchain. The authors do not specify the communication protocols used.

Li et al. [37] proposed a blockchain architecture for managing transactions in microgrids. The authors do not address user-to-user transactions, so it is not possible to analyze each user’s data separately. The blockchain is a distribution operator between microgrids. The authors only specified that a private Ethereum blockchain developed in the Go language was used, without addressing the data privacy concepts or communication protocols involved.

Niu and Zhang [38] describe a blockchain system for power distribution networks. The authors applied data compression with the blockchain Delegated Proof of Stake (DPOS) algorithm for storage on resource-limited nodes; these nodes can verify transactions by themselves. This proposal improves the efficiency of block generation and validation in the network. The work does not address the privacy or communication protocol used. The authors used a private blockchain but do not specify which. However, data compression has proven to be an effective method for storing the blockchain on devices with limited storage capacities.

Vashista and Barbhuiya [39] show a blockchain architecture where each machine and user has its address for direct communication with services. The system was implemented on the Ethereum platform using the Solidity language. The authors used the IPFS distributed data storage protocol. The IPFS stores the billing document, and the blockchain stores the IPFS hash. The authors did not show why they were using the Ethereum platform, but report that the tests were developed on a private network. The work superficially addresses privacy, only storing bill documents through IPFS. The authors do not describe which communication protocol is used.

Li et al. [40] proposed a layered architecture for a grid, edge, and cloud. Each layer performs data analysis for quick response to users. The blockchain is above these layers using dynamic pricing and smart contracts to maintain smart grid stability and allowing users to monitor details of their production and power consumption. The authors chose the Ethereum platform because it allows for more freedom to work with smart contracts and has better stability. They used a private network for prototyping, but they do not address data privacy or the communication protocols used in the blockchain.

Table 1 shows a comparison synthesis of the related works. The table is divided into eight columns, as can be seen below.

1. Reference;
2. Year;
3. Address privacy;
4. Blockchain type;
5. Blockchain used in implementation;
6. Communication protocol.

The works of Li [37], Vasistha [39], and Li [40] used the Ethereum platform. Considering this, the use of the Ethereum blockchain is most appropriate due to the integration of smart contracts and network robustness. It is not necessary to develop a specific mainchain for the SG scenario, considering that sidechains can solve the problem presented. Considering the use of private blockchains, all related works used private blockchains. The problem regarding the use of private blockchains is that only users who have access authorization can join in the blockchain. In this way, scalable solutions become infeasible for SG scenarios. Four related works address the privacy issue. In [34], different blockchains and pseudonymization are used to guarantee privacy through a group of users. In [35], the authors stored the data on the device itself, and for sharing, they used encrypted communication. None of the related work presented shows in detail the use of communication protocols and how this can change the operation of blockchain architecture for SG scenarios. Our work uses the OSGP protocol to guarantee the generalization of the developed application. A sidechain was also used to enable the scalability of the system. We also consider that privacy is an essential point in SG applications, so our solution guarantees different privacy policies according to user preferences.

## 4. Methodology and Architecture

The objective of this research was to build a possible response or solution to a problem. In this case, the problem addressed is that of making an efficient SG system using blockchains. To define the ideal solution to the problem, we compared diverse state-of-the-art solutions.

As observed in the state-of-the-art solutions, we noticed that there is a research gap regarding the use of sidechains in SG systems. There is no definitive answer to this problem concerning privacy, scalability, and universality of existing solutions.

Our solution is a sidechain architecture that is built up of three different blockchains, named BlockPRI, BlockSEC, and BlockTST. BlockPRI stores each user’s privacy preferences. BlockSEC stores the users’ data. Finally, BlockTST manages and validates information regarding the energy trade between consumers/prosumers and consumers/companies.

We developed an architecture using the three blockchains to validate the proposed idea. The Loom Network [41], a sidechain testing platform, was used to develop each blockchain. Three layers identify the proposed architecture to abstract each part of it. Table 2 shows the acronyms used to identify each device shown in the architecture.

Our architecture ensures system privacy, security, and reliability through the use of three distinct blockchains, one for each feature mentioned. This choice was made considering that it is easier to handle each of the requirements using individual blockchains. We also consider that in future applications which need to meet the same requirements (privacy, security, and trust), even in different scenarios, the three developed blockchains can integrate other applications’ contracts and data storage. However, to perform the communication between the blockchains, a federation is used to establish the connection.

The Loom Platform uses Delegated Proof of Stake (DPOS) as a consensus algorithm. According to [42], this algorithm allows the token holders to elect witnesses. Witnesses act as validators of the blockchain, proposing blocks and verifying that transactions are correct. This kind of consensus can also punish validators who try to forge corrupted blocks. To ensure democracy in the voting process, even considering that the ECs have a more significant stake of coins, the DPOS algorithm allows for the configuration of the required amount of coins to participate in the voting, allowing all network users to participate in the process.

**Figure 4:**
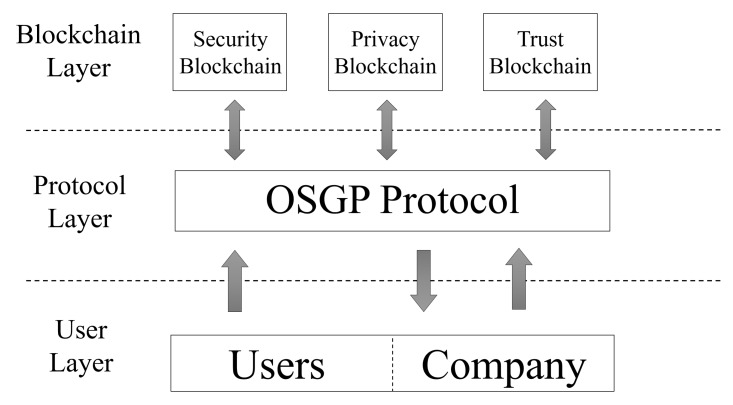
Proposed Architecture.

Three layers divide our architecture, and each layer has a specific function on each system requirement. The first layer is called the user layer. In this layer, the information is obtained and presented to users. The second layer is the protocol layer, the function of which is to define the data according to the OSGP protocol. The third layer is called the blockchain layer. This layer manages the data obtained at the protocol layer. Figure 4 illustrates the described model.

The User Layer (UL) registers users in the blockchain through a User Interface (UI). When a user wants to join the system, the user registers his SM through the UI (if the SM is not compatible, it is not possible to register). The UI is also used to make interactions with the blockchain, such as changing privacy preferences, requesting ET, verifying SM information in real time, among others.

The Protocol Layer (PL) uses the OSGP protocol to obtain and model the data package. According to [43], three standards defines the OSGP protocol. ETSI TS 103 98 rules the physical layer, ISO/IEC 14908.1defines the network layer, and finally, ETSI GS OSG 001 standard corresponds to the application layer. In our architecture, we only used the ETSI GS OSG 001 standard. This standard was used to define the data packet stored in the security blockchain. Considering this, applications running the ISO/IEC 14908.1 standard have compatibility to use our architecture. It is necessary to mention that the OSGP protocol is compatible with other SG protocols (i.e., G3-PLC and DLMS/COSEM), thus enabling the use of this architecture in other protocols as well.

**Figure A1:**
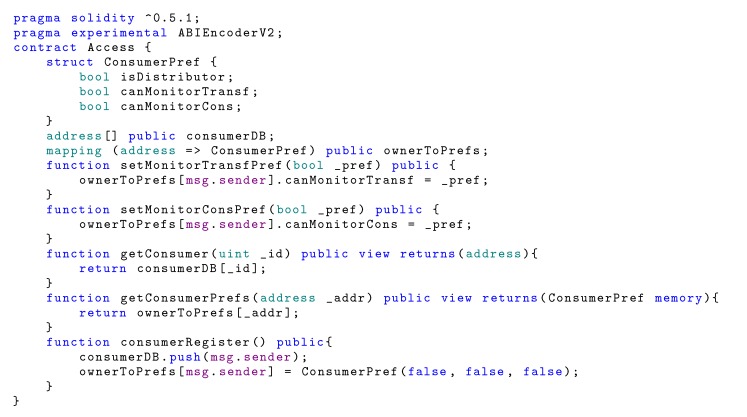
Privacy Preference Contract.

The Blockchain Layer (BL) is responsible for ensuring privacy, security, and trust in the system. BlockPRI stores smart contracts with the privacy preferences of each user; these preferences are defined by the user when registering on the network. Considering that SC is immutable when a user registers a contract in the network with his privacy preferences, this same contract can be changed only by the user who created it. The privacy SC it is called the Privacy Preference Contract (PPC). Figure A1 illustrates the code used for the situation described.

As can be seen in Figure A1, the setMonitorTransfPref() and setMonitorConsPref() functions have a bool value to define the privacy preference for monitoring ET and consumption data. The getConsumer() function returns the consumer’s address, and this should be done to verify that the person who is trying to change the SC is the owner. The getConsumerPrefs() function returns the user or concessionaire the privacy preferences of a user. Finally, the consumerRegister() function registers the user in the blockchain on first access.

**Figure A2:**
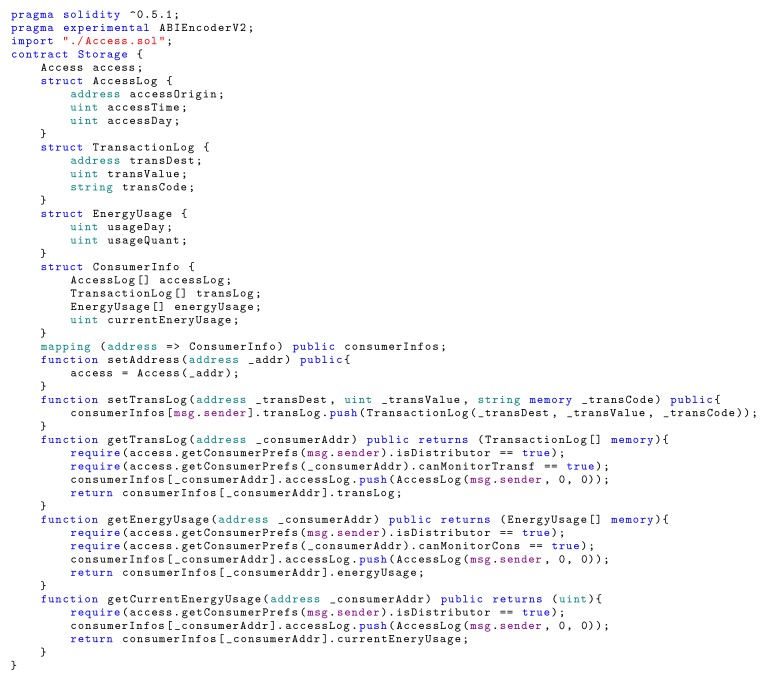
Storage Contract.

BlockSEC is responsible for storing user information. The blockchain stores data about energy usage, transactions, and other information. Whenever the Electric Company (EC) needs to store a user’s information, it must connect to BlockPRI and verify if the monitored user allows the EC to store such data (i.e., the user can define that the EC stores data of energy usage but not of transactions made with other users). However, BlockSEC only deals with the storage issue, so the EC can monitor the data in real time to have control over the network even if it does not store it. Figure A2 illustrates the SC used in BlockSEC.

As described in Figure A2, the setAddress() function checks if the preferences stored in the PPC allow for the monitoring of the user. If the user has enabled the option to be monitored, the setTransLog(), setEnergyUsage(), and setCEnergyUsage() functions store the monitored data in BlockSEC. The getTransLog(), getEnergyUsage(), and getCEnergyUsage() functions retrieve the data stored in the blockchain.

**Figure A3:**
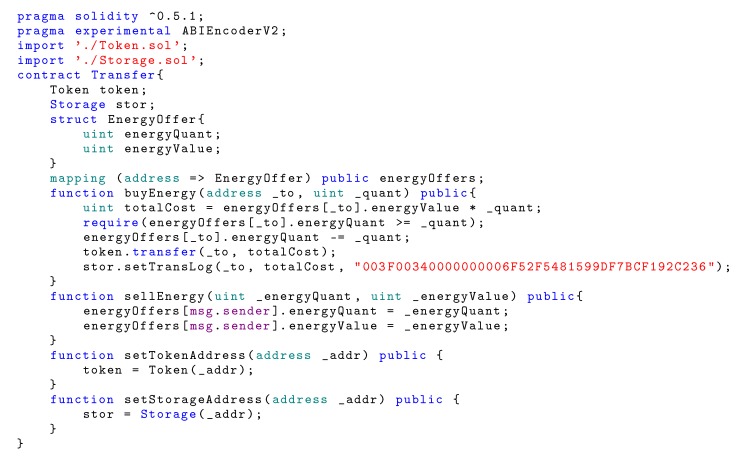
Energy transfer Contract.

BlockTST validates transactions between users. This blockchain uses smart contracts to ensure payments through the token created. The SC confirms that the token is paid only at the time the power trade is confirmed. Energy commerce can be made between Consumer/Prosumer (CP) and Consumer/Company (CC), thus allowing for a dynamic and efficient system of energy trade. Figure A3 illustrates the operation of BlockTST SC.

Figure A3 illustrates the contract of ET. The functions buyEnergy() and sellEnergy() are responsible for the process of buying and selling electricity, linking buyers and sellers through the parameters of each function. The setStorageAddress() function sends the data to the storage contract to store the transaction information in BlockSEC.

**Figure 5:**
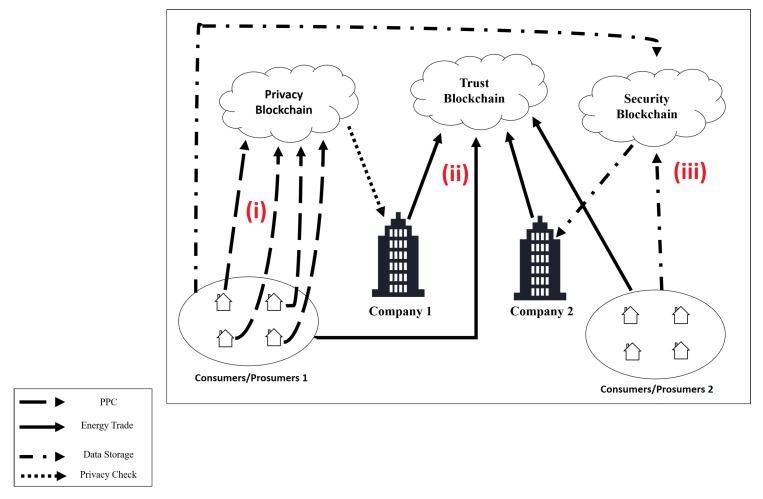
Application Scenario.

Figure 5 illustrates the operation of the proposed architecture in different situations. The illustrated scenario consists of the integration of different SG zones and the connection of different environments through the proposed architecture. To exemplify the use of the architecture, different application scenarios are used, such as (i) privacy preference register, (ii) energy trade, (iii) and monitoring.

**Figure 6:**
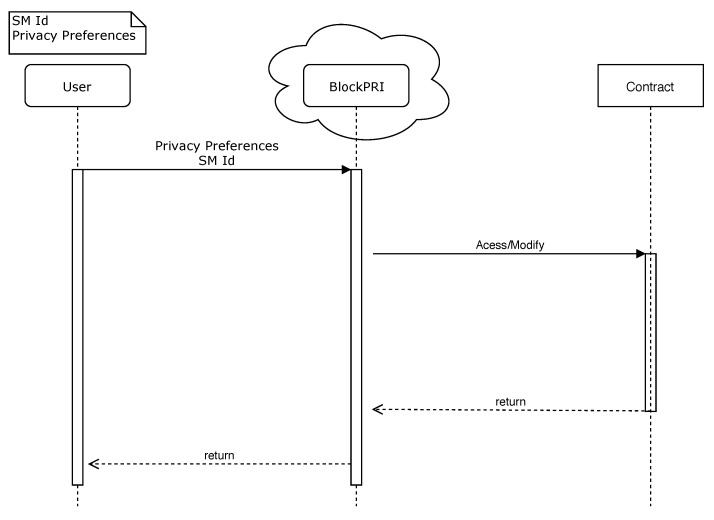
Privacy preferences registering.

In Situation (i), a user registers his SM in the system through an UI. The system registers a wallet address for each SM. At this moment, the user sets his privacy preferences. The privacy preference information is registered on the PPC and stored in BlockPRI. Only the contract owner can change the privacy preferences defined, as illustrated in Figure 6.

**Figure 7:**
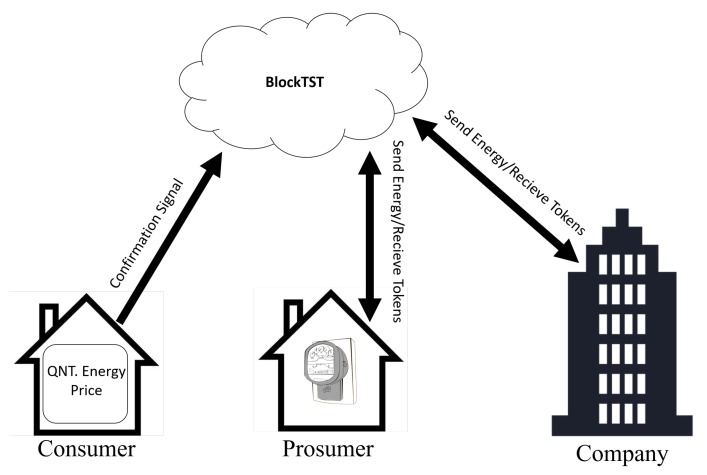
Energy trade situation.

In Situation (ii), the Energy Trade (ET) between CP and CC is illustrated. The user who purchases the electric power sets in the UI the amount of energy to be purchased and the price to be paid. When the ET happens, the SM sends a confirmation signal to BlockTST through the UI, so the SC can validate the trade process and give the tokens to the power seller. The same process occurs for ET between the electrical company and the user. Figure 7 illustrates the described situation.

**Figure 8:**
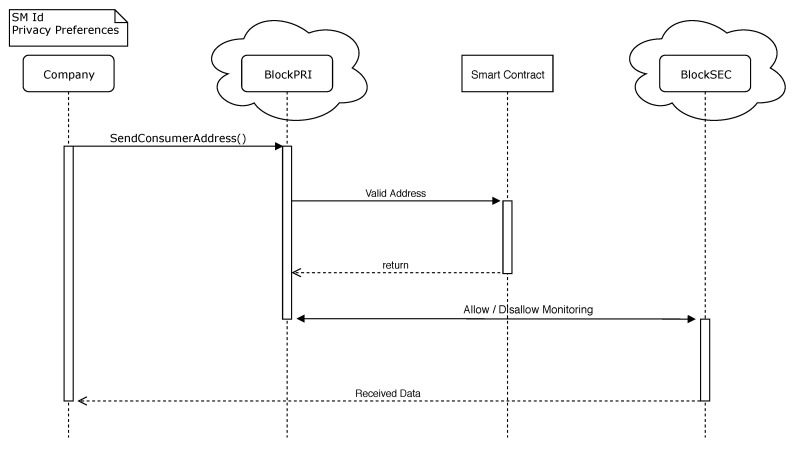
Energy trade situation.

In Situation (iii), the EC monitors a user. When this happens, the company connects to BlockPRI and gives the address of the user who it wants to monitor. If the user’s PPC allows monitoring, BlockSEC stores the monitored data in a private blockchain that only the EC has access to. If a user suspects that it has been monitored in an unauthorized way, that user may request a court order to verify BlockSEC, and considering that information cannot be deleted from the blockchain, the system is entirely auditable. Figure 8 shows the described situation in detail.

To validate the proposed architecture, we performed performance, safety, and efficiency tests on the system in the described scenarios. The tests aim to prove the feasibility of the implementation of our architecture.

## 5. Tests and Results

We used the Loom Platform to perform the tests and obtain the results. Loom is a platform focused on the development of sidechain applications. The results obtained are related to the number of transactions processed by the network, the contract costs, a comparison of the energy price purchased by the blockchain in terms of the purchase of conventional energy, the adjustment of the energy price according to the PPC, the system response to unauthorized monitoring attempts, and BlockSEC’s data structure view when monitoring a user. All of the tests were performed on a Kubuntu OS 18.04 notebook with an Intel Core i5-8625U @ 1.60 GHz processor and 8 GB of RAM.

### 5.1. Transaction Processing Time

**Figure 9:**
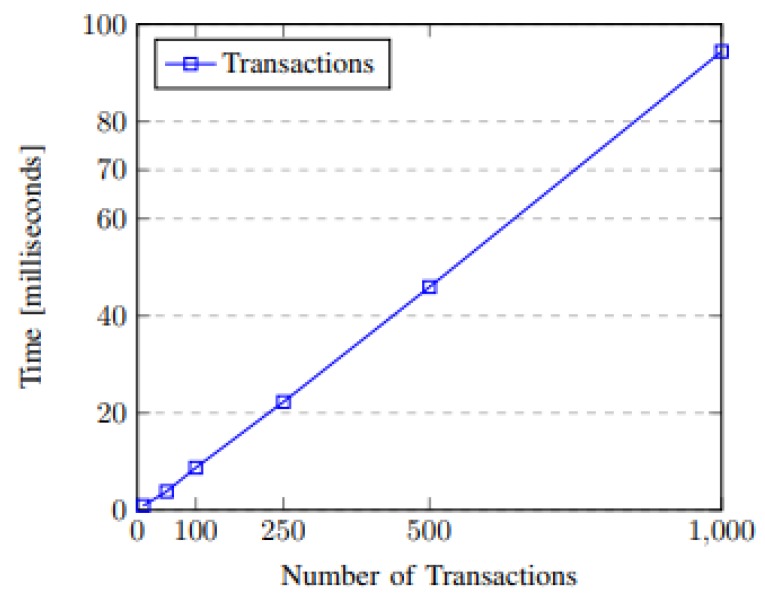
Transactions per second.

Figure 9 shows the processing time spent on each set of transactions performed. The samples chosen for the test were 10, 50, 100, 250, 500, and 1000 transactions. The linear growth corresponds to the behavior expected within a stress test of the system, ranging from 0.8 to 94 s of the processing time. The environment used for testing relied on immediate mining, and we discarded any additional waiting time that would interfere with the results. The data size used in this test was 144 bytes; this corresponds to the OSGP protocol data packet size.

### 5.2. Token Cost with Different Privacy Settings

Table 3 shows four situations based on different levels of demand, generation, privacy preferences, and type of trade. The types of possible trades are between CP or CC. The amount of energy used is the same for each of them, for ease of comparison. In Situations 1 and 3, where consumption (or demand) is high, the Smart Grid Token (SGT) values involved in the exchange tended to be higher as a result of the shortage of the product. Considering that the consumer allowed themselves to be monitored was unimportant because it is a relationship of trade between consumers, neither is allowed to monitor the other, with no benefits to any party. In Situations 2 and 4, however, we can observe two aspects that make the price involved in the transaction cheaper. The first is high supply and low demand, denoting excess production. The second is the importance of monitoring when in an exchange with a dealership. In Situation 2, when dealing specifically with this aspect, there is no difference in price, since the privacy options are disabled, but in Situation 4, having the options enabled made it possible to make the transaction cheaper. In Situations 5 and 6, a Prosumer/Company (PP) trade is performed, with the privacy options enabled, the prosumer can sell his energy to the company for a higher price (this is used to incentivize the prosumer to share his information with the companies).

### 5.3. Smart Contract Cost

Table 4 shows the deployment cost of each contract developed. We used the Ropsten TestNet to evaluate all contracts. The storage contract, which is responsible for storing information and is the most expensive functionality in a blockchain, had the highest cost in ETH. The other contracts have a lower cost because the functions used in each of them do not have the main purpose of storing data, but access control.

In Table 5, we show the relationship of a function to its contract and the cost of operation. As can be seen, the predominance of the most expensive ones is almost entirely from storage class functions, since they are blockchain write operations (even getters, since the use of functions results in saving the corresponding get-log operation to the address). Transfer functions also had some cost, even if less excessive.

### 5.4. Privacy Violation Test

**Figure 10:**
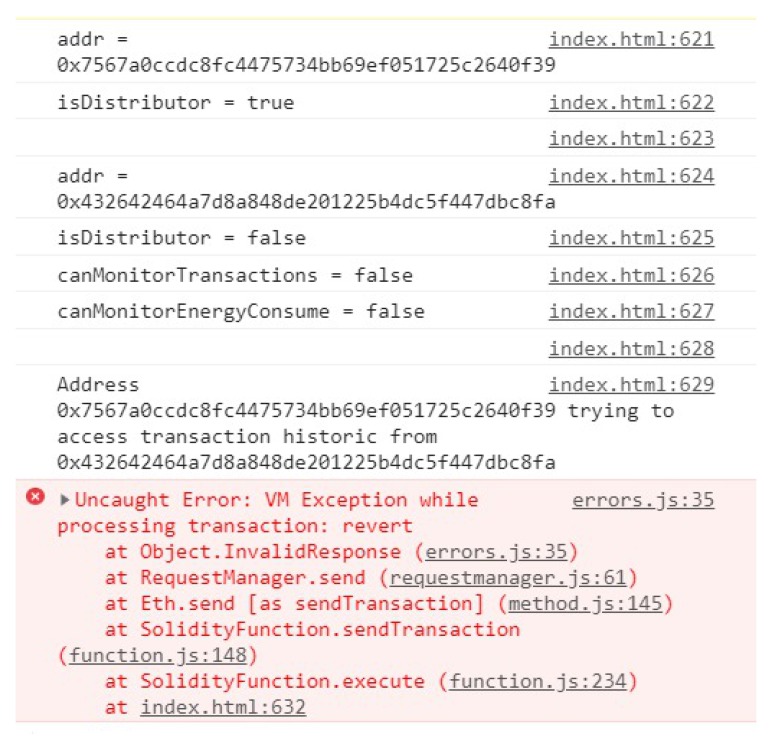
Unauthorized access to storage.

In Figure 10, we demonstrate, in the console, the result of an attempt at unauthorized access to the data in the storage. First, we define an address as being an electrical company to try to access. Then, another address is registered and stored on BlockPRI, with the distributor mode disabled, as well as the settings that allow access to data stored in the blockchain. Finally, we had the distributor address try to access the historical data of the consumer who chose to protect himself. The result of the procedure was an error coming directly from the execution of the operation, whose implementation takes care of interrupting it if the request made is not by the client’s privacy options.

### 5.5. Data Structure Stored in BlockSEC

**Figure 11:**
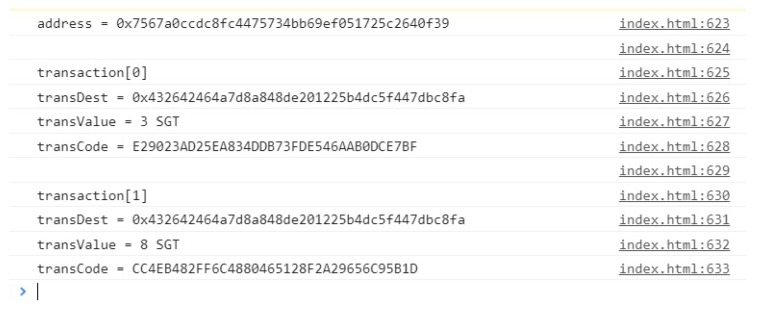
Stored Contents.

Figure 11 shows one of the data structures inside the data storage blockchain. This structure is a shared vision for the concessionaires whose consumers have allowed their data to be monitered. All transactions performed by an address are stored in the same structure, with the destination address, transaction value (in SGT), and the table encrypted and formatted within the specifications of the OSGP model. We can view the transaction history of a particular consumer, with all the security and standardization found in the current state-of-the-art.

## 6. Conclusions

Our work presented a blockchain architecture for SG systems using sidechains. Our architecture ensures privacy, security, and trust in the system through the use of three distinct blockchains. Our architecture also guarantees universality through the use of the OSGP protocol, since it ensures a universal architecture for SG environments.

For transaction processing time tests, the results illustrate a linear growth. The processing time was adequate for the current scenario, considering that 1000 transactions were processed in approximately 94 ms. For the SGT token created, we realized that the smart contract architecture provided a price decrease in consumer–company transactions for users who chose to share their information. Regarding the cost of deploying the contract, the most expensive contract was the storage contract, this result was expected as storage is the most costly operation on a blockchain. The privacy test developed showed that the privacy preferences stored in smart contracts are met; this was proven through an interaction in which an attempt was made to access data not allowed by the contract preferences. Finally, concerning the data structure stored at BlockSEC, it can be observed that the stored data illustrates the information required for the utility when a user allows for it to be monitored.

With the tests developed, we realized that the proposed architecture is feasible for use in real scenarios. Using different blockchains for each system requirement allowed us to ensure privacy, security, and trust holistically. To stimulate users to share their data with the electric company, a system of price discounts on the purchase of electricity is given to users who share their data to electrical companies. However, as the system grows, the processing time of transactions may increase and generate performance problems that must be treated in the future. We also mention that BlockSEC, which stores user data, may need a large storage capacity to be able to hold all the data.

The developed architecture meets the requirements presented in the introduction of this work. The developed solution presented has its differential based on state-of-the-art solutions using sidechains in smart grid systems. Thus, we conclude that the proposed work presents a scientific and technical contribution, proposing a different approach for the use of blockchains in smart grid systems.

For future work, we suggest performance studies in communication between blockchains. We also suggest studies focused on the DPOS consensus algorithm to optimize democratization in validator voting and on the issue of transaction processing time and data storage in BlockSEC, seeking to optimize these functionalities to ensure a completely efficient system.

## Figures and Tables

**Table 1:** Related Work.

Work	Year	Privacy	Blockchain Type	Platform Used	Communication Protocol
Guan et al. [34]	2018	Yes	Private	NS	NS
Gür et al. [35]	2019	Yes	Private	Hyperledger	NS
Gai et al. [36]	2019	Yes	Private	Hyperledger	NS
Li et al. [37]	2019	No	Private	Ethereum	NS
Niu and Zhang et al. [38]	2019	No	Private	NS	NS
Vashista and Barbhuiya [39]	2019	Yes	Private	Ethereum	NS
Li et al. [40]	2019	No	Private	Ethereum	NS
Our work	2020	Yes	Sidechain	Ethereum	OSGP

NS: Not Specified; OSGP: Open Smart Grid Protocol.

**Table 2:** Acronym Definition.

Acronym	Definition
PPC	Privacy Preference Contract
BL	Blockchain Layer
PL	Protocol Layer
UL	User Layer
EC	Electric Company
CP	Consumer/Prosumer
CC	Consumer/Company
PP	Prosumer/Company
UI	User Interface
ET	Energy Trade
EC	Electrical Company

**Table 3:** Token Cost in Different Situations.

	Demand	Generation	Privacy Preferences	Trade Type	Energy Ammount	Price (SGT)
Situation 1	75	25	Disabled	CP	50	30
Situation 2	25	75	Disabled	CC	50	20
Situation 3	75	25	Enabled	CP	50	30
Situation 4	25	75	Enabled	CC	50	10
Situation 5	25	75	Enabled	PP	50	15
Situation 6	25	75	Disabled	PP	50	10

**Table 4:** Deploy Cost by Contract.

Contract	Cost (ETH)
Access	0.001413
Storage	0.003389
Transfer	0.001417
Token	0.001862

**Table 5:** Function Cost.

Function	Contract	Cost (ETH)
setTranslog	Storage	0.000821
buyEnergy	Transfer	0.000536
getEnergyUsage	Storage	0.000519
getTranslog	Storage	0.000447
sellEnergy	Transfer	0.000452

## Appendix A

Figure A1 illustrates the PPC. This contract is responsible for storing the privacy preferences of each user. The algorithm is described detailed in Section 4.

## Appendix B

Figure A2 illustrates the storage SC. This contract is responsible for checking the preferences stored in the PPC and performing an action based on these preferences. The algorithm is described detailed in Section 4.

## Appendix C

Figure A3 illustrates the energy transfer SC. This SC is responsible for validating the energy trade between users. The algorithm is described detailed in Section 4.
